# Covariates of a healthy diet and physical activity self-management one year after Bariatric surgery: A cross-sectional study

**DOI:** 10.1371/journal.pone.0287137

**Published:** 2023-10-18

**Authors:** Maryam Maghsoodlo, Elham Shakibazadeh, Maryam Barzin, Yahya Salimi, Zeinab Mokhtari, Mehdi Yaseri

**Affiliations:** 1 Department of Health Education and Promotion, School of Public Health, Tehran University of Medical Sciences, Tehran, Iran; 2 Research Institute for Endocrine Sciences, Obesity Research Center, Shahid Beheshti University of Medical Sciences, Tehran, Iran; 3 Social Development & Health Promotion Research Center, Health Institute, Kermanshah University of Medical Sciences, Kermanshah, Iran; 4 Nutrition and Food Security Research Center, Isfahan University of Medical Sciences, Isfahan, Iran; 5 Department of Epidemiology and Biostatistics, Tehran University of Medical Sciences (TUMS), Tehran, Iran; Shahid Beheshti University of Medical Sciences School of Dentistry, ISLAMIC REPUBLIC OF IRAN

## Abstract

**Background:**

Healthy diet and physical activity self-management is important in maintaining weight loss and preventing weight regain after bariatric surgery. We aimed at evaluating covariates of healthy diet and physical activity self-management among patients undergone bariatric surgery using Health Action Process Approach (HAPA) model.

**Method:**

In this cross-sectional study, 272 patients with a history of bariatric surgery were selected from the data registry of Tehran Obesity Treatment Study (TOTS). Data were collected using bariatric surgery self-management standard questionnaire (BSSQ), and items based on HAPA model for healthy diet and physical activity self-management. Data were analyzed using Path analysis and AMOS version 24.

**Results:**

The mean score of self-management was (32 ± 10SD). Coping planning construct (β = 0.22; p<0.001) and risk perception (β = 0.02; p<0.01) in dietary self-management and action planning (β = 0.16; p = 0.001) and risk perception (β = 0.001; p = 0.17) in physical activity self-management had the highest and lowest effect powers, respectively. Coping planning (β = 0.22; p<0.001) and action planning (β = 0.17; p<0.03) in diet, and action planning (β = 0.16; p = 0.010) in physical activity were significantly related to self-management. Also, task-coping self-efficacy (β = 0.28; and p<0.001), outcome expectancies (β = 0.37; p<0.001), risk perception (β = 0.13; p = 0.015) in diet and coping self-efficacy (β = 0.50; p<0.001), outcome expectancies (β = 0.12; p = 0.021) in physical activity were significantly related to behavioral intention. The values of CFI = 0.939 and RMSEA = 0.052 for diet and CFI = 0.948 and RMSEA = 0.048 for physical activity indicated adequate fit.

**Conclusion:**

HAPA was applicable as a framework for interventions promoting healthy diet and physical activity self-management in patients who have undergone bariatric surgery.

## Introduction

Severe obesity, a growing health condition worldwide [1], is associated with medical conditions such as type 2 diabetes, hypertension, and dyslipidemia [2]. The age-standardized prevalence of obesity has increased from 4.6% in 1980 to 14.0% in 2019 [3]. The trends of severe obesity have elevated considerably in Africa and the Middle East [4].

Bariatric surgery is currently the main treatment strategy for severe obesity [5]. Research studies show that weight regain and the reappearance of type 2 diabetes and other diseases are major concerns after bariatric surgery [6–9]. Some studies reported that 20–24% of patients gained more than 15% of their body weight after bariatric surgery [10–12].

A range of behavioral, dietary, psychological, physical, and medical considerations can play a role in suboptimal long-term weight loss [13]. Several studies have assessed the lifelong adherence to a healthy lifestyle after the surgery and found that non-adherence with dietary recommendations/loss of dietary control and exercise recommendations were important causes for weight regain post-surgery [14–19]. Several studies have shown that self-management is the best approach to maintaining weight loss (WL) and preventing weight gain after bariatric surgery [20–22]. The critical components of self-management after bariatric surgery to maintain WL are healthy eating behaviors and regular physical activity [23,24]. Wouters, Lent et al. discussed the benefits and obstacles of physical activity after bariatric surgery regarding the importance of self-management and following a healthy diet [25,26]. Lifestyle changes are also important to obtain and maintain optimal WL after bariatric surgery [8]. However, there is no recommendation about the type of postoperative psychological interventions and their optimal timing concerning surgery [2]. Scientific evidence highlights the necessity of designing and implementing health education and behavior change programs to reduce the barriers to adherence among patients following bariatric surgery.

The first step in the planning process of any health education program is selecting an appropriate behavior change theory/model through which the program is directed scientifically and kept on the right path [27,28]. Health Action Process Approach (HAPA) has been proposed by Schwarzer in 2008. Motivational and voluntary parts of the approach are two constructs of this model. The motivational part includes risk perception, outcome expectancies, task self-efficacy, and behavioral intentions; and the voluntary part includes action planning, coping planning, coping self-efficacy, and recovery self-efficacy [29]. This model emphasizes on the stabilization of behavior and is effective for long-term behaviors such as physical activity, nutritional behaviors, and preventive diet [29–31]. There are no studies available that evaluate self-management of diet and physical activity in patients with bariatric surgery using HAPA. This study aimed to assess healthy diet and physical activity self-management and their covariates among patients undergoing bariatric surgery in Tehran Obesity Treatment Study (TOTS).

## Methods and materials

This cross-sectional study was part of a Ph.D. thesis approved by the Ethics Committee at Tehran University of Medical Sciences (IR.TUMS.SPH.REC.1400.230). The study population consisted of 300 patients registered at Hakim Obesity Clinic in Tehran who had undergone bariatric surgery at least one year ago from November 2021 to March 2021–2022. The inclusion criteria included patients who had undergone bariatric surgery at least one year before and were willing to participate in the study. Research aims were explained to the participants and after obtaining the informed consent, they completed the questionnaires. Confidentiality was ensured.

We needed to have at least 217 samples to obtain a minimum precision of 2 for 95% confidence assuming a standard deviation of 15 based on the following formula

$$n=\frac{{\left(Z_{1-\alpha /2}\right)}^{2}\times \left(S^{2}\right)}{d^{2}}$$

The total score TOTALBSSQ was obtained from the sum of the Likert scores of the questionnaire items, each of which was between 0 and 2.

### Study measures

The questionnaire had three parts: 1) demographic items including age, gender, level of education, economic status, and marital status; 2) items based on HAPA for a healthy diet and physical activity; and 3) bariatric surgery self-management standard questionnaire (BSSQ).

To design HAPA items, we reviewed the existing questionnaires regarding factors affecting self-management of a healthy diet and physical activity; and also reviewed the existing standard questionnaires on HAPA for other health issues [32–35]. Then we placed the identified items into the constructs of HAPA to provide a preliminary tool with certain HAPA constructs. We went through the process of designing items, including face validity, content validity ratio (CVR), and content validity index (CVI). Reliability was assessed using Cronbach’s alpha (0.7–0.95 for different constructs), and the intra-cluster correlation coefficient index (0.7–0.91). The healthy diet section of the questionnaire included seven constructs and 23 items, including task and coping self-efficacy (3 items), action planning (3 items), coping planning (4 items), recovery self-efficacy (3 items), risk perception (4 items), outcome expectancies (4 items), and behavioral intention (2 items). The physical activity section had seven constructs and 22 items, including task and coping self-efficacy (3 items), action planning (4 items), coping planning (3 items), recovery self-efficacy (3 items), risk perception (3 items), outcome expectancies (4 items), and behavioral intention (2 items) (Table 1).

**Table 1:** The Cronbach’s alpha and correlation coefficients according to the constructs of HAPA.

Healthy diet	Cronbach’s Alpha	ICC	Physical activity	Cronbach’s Alpha	ICC
Coping self-efficacy	0.79	0.79	Coping self-efficacy	0.87	0.81
Task self-efficacy	0.77	0.74	Task self-efficacy	0.89	0.90
Recovery self-efficacy	0.71	0.75	Recovery self-efficacy	0.91	0.85
Coping planning	0.74	0.85	Coping planning	0.74	0.88
Action planning	0.72	0.83	Action planning	0.95	0.91
Risk perception	0.75	0.71	Risk perception	0.77	0.72
Outcome expectancies	0.81	0.80	Outcome expectancies	0.77	0.71
Behavioral intentions	0.91	0.90	Behavioral intentions	0.72	0.72

BSSQ was designed and assessed by Welch et al. (2008). This questionnaire includes 32 items with seven behavioral domains of eating, drinking, protein consumption, physical activity, management of dumping syndrome, eating fruits, vegetables, and whole grains, and taking vitamin and mineral supplements [17]. Amini et al. assessed the questionnaire’s validity and reliability in Persian language [36].

### Data analysis

Path analysis using AMOS 24 was used to evaluate the cause-effect relationship and to determine the strength of the constructs’ effect on a healthy diet, physical activity, and the relationship between the constructs. Also, T-test, analysis of variance, was used to assess the relation of the demographic variables with total BSSQ score and HAPA constructs. To assess the simultaneous effect of this demographic variate on total BSSQ score General Lwasr Model was applied. Also, these relations with HAPA constructs were tested using the MANCOVA (Multivariate Analysis of Covariance). The level of significance in the tests was considered 0.05.

When the initial questionnaires were filled, if a question was not answered, the patients were asked to fill it during the follow-up and re-request, so we did not have any data loss in this study.

## Results

A total of 272 out of 300 participants completed the questionnaires. The mean and standard deviation of the BSSQ total score was 32.2±10.1 (CI: 95% 31.0 TO 33.4). The mean age (SD) of the participants was 32 (6.3) years. Most participants were women (76.1%), had a bachelor’s degree (41.9%), were married (67.3%), and had reported a moderate economic status (75.4%) (Table 2).

**Table 2:** Characteristics of study participants (n = 272).

Characteristics	Levels	Statistics	Value
Gender	Female	N(%)	**207 (76.1)**
	Male	N(%)	**65 (23.9)**
Age	mean± (SD)		**32± 6.3**
Education	Illiterate	N(%)	**5 (1.8)**
	Diploma	N(%)	**96 (35.3)**
	Bachelor’s degree	N(%)	**114 (41.9)**
	Postgraduate	N(%)	**57 (21.0)**
Marital status	Single	N(%)	**87 (32.0)**
	Married	N(%)	**183 (67.3)**
Economic status (Self-report)	Weak	N(%)	**12 (4.4)**
	Moderate	N(%)	**205 (75.4)**
	Good	N(%)	**55 (20.2)**

**Fig 1:**
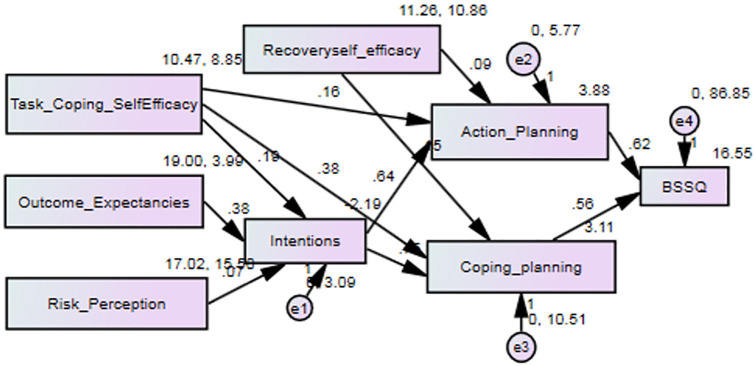
Path analysis for healthy diet self-management showed that in a healthy diet, self-management, task and coping self-efficacy, outcome expectancies, and risk perception were significantly related to the behavioral intention of a healthy diet.

The effect of independent variables on a healthy diet and physical activity self-management was assessed using path analysis to identify variables affecting the patients’ self-management regarding a healthy diet and physical activity. The highest and lowest power of significant effect in healthy diet self-management was related to coping planning and risk perception constructs. However, all constructs tended to enhance self-management significantly (Table 3). Fig 1 shows the path analysis for healthy diet self-management.

**Table 3:** The results of the power of effect of the effective factors on healthy diet self-management.

Variables	Dependent Variable	Effect size	P-value*
Coping planning	self-management	0.223	P<0.001
Action planning		0.178	P = 0.003
Behavioral intention		0.167	P<0.001
Task and coping self-efficacy		0.143	P<0.001
Outcome expectancies		0.062	P<0.001
Recovery self-efficacy		0.047	P = 0.032
Risk perception		0.022	P = 0.015

* Based on path analysis.

**Fig 2:**
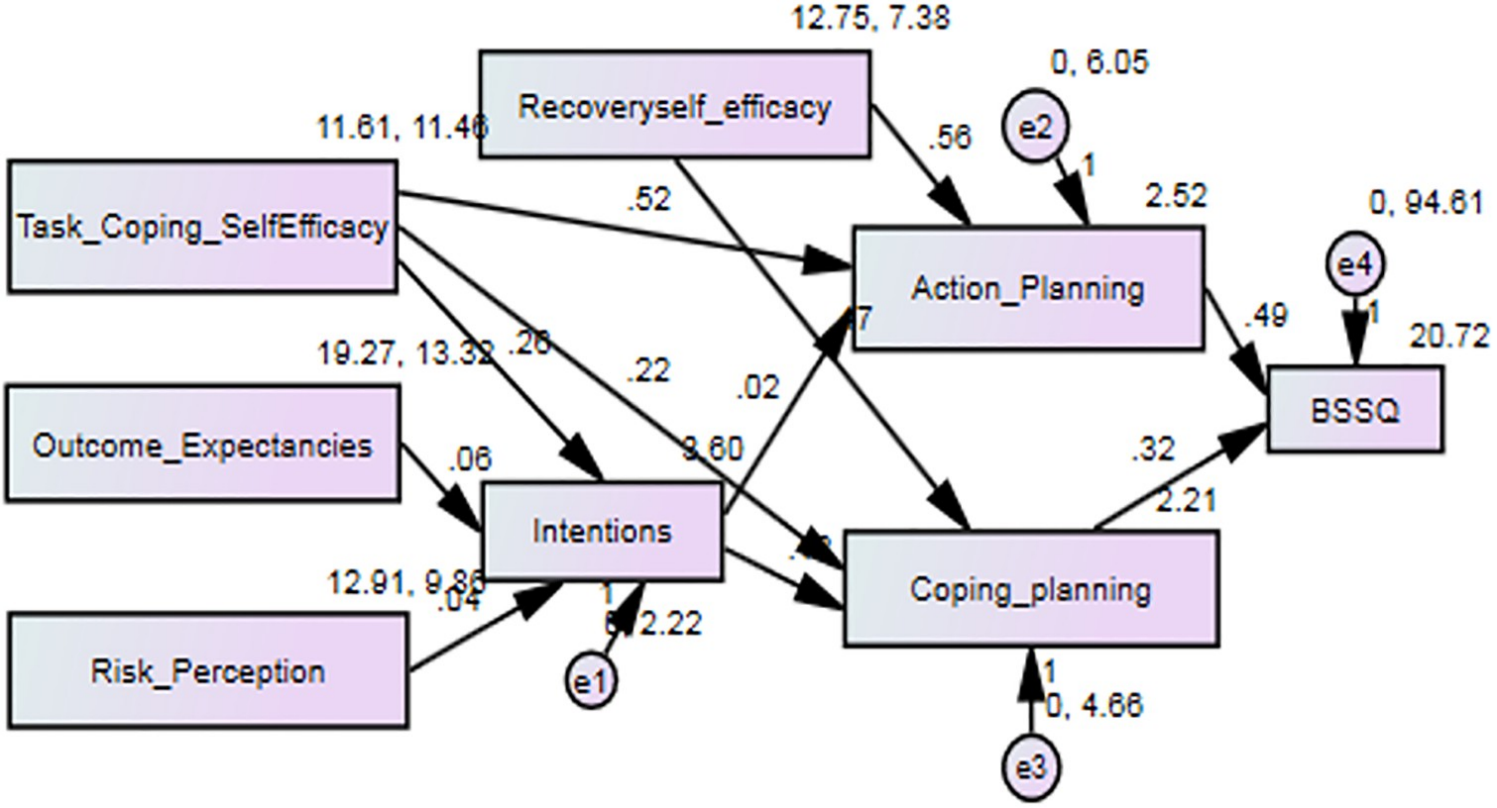
Path analysis for physical activity self-management and coping self-efficacy and outcome expectancies were significantly related to physical activity intention.

Regarding physical activity self-management, the highest and lowest power of the effect was related to action planning and risk perception constructs, respectively (Table 4). Fig 2 shows the path analysis for self-management of physical activity.task

**Table 4:** The results of the power of effect of the effective factors on physical activity self-management.

Variables	Dependent Variable	Effect size	P-value*
Action planning	Self-management	0.167	P = 0.01
Task and coping self-efficacy		0.115	P<0.001
Recovery self-efficacy		0.115	P<0.001
Coping planning		0.085	P = 0.085
Behavioral intention		0.008	P = 0.008
Outcome expectancies		0.001	P = 0.001
Risk perception		0.001	P = 0.001

* Based on Path analysis.

The results of our study showed that in a healthy diet, self-management, task and coping self-efficacy (β = 0.28; p<0.001), outcome expectancies (β = 0.37; p<0.001), and risk perception (β = 0.13; p = 0.015) were significantly related to the behavioral intention of a healthy diet. Task and coping self-efficacy (β = 0.17; p = 0.001), behavioral intention (β = 0.45; p<0.001), and recovery self-efficacy (β = 0.11; p<0.032) were significantly related to action planning of diet. Task-coping self-efficacy (β = 0.29; p<0.001), behavioral intention (β = 0.38; p<0.001), and recovery self-efficacy (β = 0.12; p<0.014) were significantly related to coping planning of diet. Coping planning (β = 0.22; p<0.001) and action planning (β = 0.17; p<0.03) were significantly related to self-management of a healthy diet.

In self-management of physical activity, task and coping self-efficacy (β = 0.50; p<0.001) and outcome expectancies (β = 0.12; p = 0.021) were significantly related to physical activity intention. Task and coping self-efficacy (β = 0.52; p<0.001) and recovery self-efficacy (β = 0.48; p<0.001) were significantly associated with physical activity coping planning. Recovery self-efficacy (β = 0.44; p<0.001) was significantly related to action planning in physical activity, and action planning (β = 0.16; p = 0.010) was significantly associated with self-management of physical activity.

There was a significant relationship between gender and self-management (p<0.05); however, this relationship lost its significance in the multivariable analysis (Table 5).

**Table 5:** Relationship between demographic variables and total BSSQ score.

Variables	Level	Mean±SD	p_ Value	P_Multivariable***
**Age** *	17–35	31.07 ± 10.09	0.27	0.66
	36–50	32.80 ± 10.33		
	51–66	33.69 ± 8.87		
**Gender** **	Male	30.04 ± 8.73	0.04	0.10
	Female	32.87 ± 10.39		
**Education** *	High school diploma	31.44 ± 10.14	0.61	0.53
	Bachelor’s degree	32.48 ± 10.14		
	Postgraduate degree	32.96 ± 9.92		
**Marital status** **	Single	30.80 ± 10.85	0.253	0.41
	Married	32.86 ± 9.65		
**Economic status** **	Poor and moderate	31.79 ± 10.15	0.19	0.29
	Good	33.78 ± 9.71		

* Based on ANOVA.

** Based on the t-test.

*** Based on the general linear model.

Table 6 shows the results of demographic variables and HAPA constructs regarding a healthy diet. In the multivariable model, the whereas statistically significant relationships between education and task and coping self-efficacy (p<0.05), gender and outcome expectancies (p = 0.006), and gender and behavioral intention (p = 0.03).

**Table 6:** Results of HAPA constructs regarding healthy diet and demographic variables.

Variable	Task and coping self-efficacy		Outcome expectancies		Risk perception		Acton planning		Coping planning		Recovery self-efficacy		Behavioral intentions	
	P-_Value_	P *** Multivariate	P-_Value_	P Multivariate	P-_Value_	P Multivariate	P-_Value_	P Multivariate	P-_Value_	P Multivariate	P-_Value_	P Multivariate	P-_Value_	P Multivariate
Age*	0.60	0.54	0.24	0.32	0.88	0.83	0.33	0.36	0.76	0.87	0.20	0.53	0.56	0.42
Gender**	0.21	0.27	0.004	0.006	0.14	0.19	0.58	0.77	0.35	0.43	0.33	0.19	0.02	0.03
Marital status**	0.78	0.76	0.74	0.78	0.94	0.94	0.56	0.54	0.43	0.45	0.39	0.68	0.61	0.53
Education*	0.04	0.05	0.30	0.15	0.29	0.31	0.05	0.03	0.14	0.11	0.26	0.49	0.22	0.23
Economic status**	0.49	0.46	0.05	0.02	0.77	0.75	0.32	0.11	0.30	0.11	0.59	0.45	0.14	0.08

* Based on ANOVA.

** Based on t-test.

*** Based on a general linear model.

Table 7 shows the results of demographic variables and HAPA constructs regarding physical activity. In the multivariable model, there were statistically significant relationships between marital status and coping planning (p = 0.03), gender and outcome expectancies (p = 0.04), gender and recovery self-efficacy (p = 0.01), and economic status and recovery self-efficacy (p = 0.04).

**Table 7:** Relationship between demographic parameters and HAPA constructs regarding physical activity.

Variable	Task and coping self-efficacy		Outcome expectancies		Risk perception		Acton planning		Coping planning		Recovery self-efficacy		Behavioral intentions	
	P-_Value_	P *** Multivariate	P-_Value_	P Multivariate	P-_Value_	P Multivariate	P-_Value_	P Multivariate	P-_Value_	P Multivariate	P-_Value_	P Multivariate	P-_Value_	P Multivariate
Age*	0.41	0.17	0.03	0.09	0.79	0.85	0.85	0.29	0.97	0.83	0.52	0.32	0.67	0.51
Gender**	0.97	0.94	0.03	0.04	0.104	0.17	0.24	0.15	0.19	0.15	0.02	0.01	0.14	0.07
Marital status*	0.76	0.34	0.51	0.94	0.89	0.88	0.21	0.03	0.72	0.66	0.82	0.508	0.19	0.11
Education*	0.19	0.205	0.71	0.708	0.11	0.102	0.50	0.42	0.003	0.009	0.39	0.71	0.47	0.54
Economic status*	0.51	0.83	0.53	0.43	0.59	0.36	0.65	0.68	0.17	0.59	0.03	0.04	0.79	0.77

* Based on ANOVA.

** Based on t-test.

*** Based on a general linear model.

## Discussion

In this cross-sectional study, we aimed to assess healthy diet and physical activity self-management among patients who had undergone bariatric surgery. Previous studies have shown that HAPA was a useful approach for measuring self-management of a healthy diet and physical activity [29,31,37,38]. The mean score of self-management indicated low adherence. It seems necessary to design and implement interventions to promote a healthy diet and physical activity self-management.

Patients who underwent bariatric surgery tended not to follow a healthy diet and did not have regular physical activity. Their lack of awareness of the complications and problems of failure to follow a healthy diet necessitates improving their perceived abilities to follow a healthy diet and increasing self-efficacy related to a healthy diet and physical activity. The results of the BSSQ self-management behavior in the Welch study showed that the mean BSSQ total adherence score over time was consistently 60–70%, indicating moderate self-management [17].

Task and coping self-efficacy, outcome expectancies, and risk perception were predictors of healthy diet behavioral intention. Also, task and coping self-efficacy had the highest beta coefficient among different predictors of behavioral intention. These findings were consistent with some previous studies in China [39] and Switzerland and England [37]. Similar to the present study, in these studies, action self-efficacy was significantly related to behavioral intention.

Action self-efficacy and outcome expectancies were significantly related to the intention of physical activity. This is in line with other studies that showed significant correlations between intention and action self-efficacy, action planning, and psychological consequences of physical activity [40–43]. The intention was supported by self-efficacy. In other words, self-efficacy was a major influencing factor that referred to a specific perceived ability to perform a desired behavior. After forming the intention, the individual enters the voluntary phase. Our study findings fully confirmed the premise of the HAPA; because among the three mentioned constructs, task, and coping self-efficacy was the strongest predictor of intention. Individuals who do not believe in their abilities to perform the desired behavior will have difficulties accepting and following that behavior. Individuals with high levels of self-efficacy envision success, anticipate the potential consequences of various strategies, and are likelier to initiate a new behavior. In contrast, people with less self-efficacy focus more on failure, doubt their abilities, and tend to postpone behavior [44]. However, only having a high level of self-efficacy is not enough to carry out and continue self-management of physical activities and a healthy diet, and it should be strengthened by using different strategies of other constructs.

Numerous studies have highlighted the prominent role of outcome expectancies in explaining dietary behaviors, which necessitates lifestyle interventions. According to the present study, the outcome expectancies construct significantly predicted behavioral intention of following a healthy diet and physical activity. Many previous studies in the field of healthy lifestyles, including nutritional behaviors and physical activity, considered outcome expectancies as the best predictor of behavioral intention following the action self-efficacy construct [38,45]. Barg et al., in their study of predictors of physical activity among inactive middle-aged women, showed that outcome expectancies had a significant and direct effect on the intention to do physical activity [46]. Similarly, Pinidiyapathirage et al. confirmed that outcome expectancies positively and significantly affected the intention [47]. Clarifying the social and physical consequences of physical activity and a healthy diet, providing emotional reflections, and strengthening interpersonal relationships could form a stronger intention to perform physical activity and follow a healthy diet in these patients.

The significant relationship between perceived risk and intention was another finding of the current study, similar to previous studies [42,46]. Perceived risk is an important motivational force for adopting healthy behaviors. Patients after surgery are more likely to face daily health threats and are motivated to maintain their health. Perhaps the main explanation is the occurrence of physical changes and the increase in the prevalence of prominent health and disease problems, which increases the feeling of vulnerability to diseases and ultimately increases the intention to adopt preventive measures [48,49].

Similar to previous studies in nutrition behaviors [37,50,51], in this study, action planning played a significant role as a mediator between intention and self-management of a healthy diet and physical activity. However, coping planning failed to act as a mediating variable between intention and self-management of physical activity. It seems that this lack of mediating role of coping planning between intention and self-management of physical activity roots from a wide range of obstacles to performing the behavior in this target group. Future studies would better understand these barriers and the role of coping planning structure in patients undergoing bariatric surgery by fully identifying them and including them in the applied tools. It should be noted that HAPA greatly impacted behavior by considering obstacles to behavior through coping planning and task and coping self-efficacy. Thus, it can be an appropriate model for understanding the beliefs of surgical patients regarding health behaviors and providing educational interventions in this field because the obstacles to performing such behaviors in post-operational patients are far more than in healthy people [52]. Further studies are suggested to evaluate the questionnaire in any bariatric surgical procedures, separately.

One of the limitations of this study was to measure the participants’ self-management to follow a healthy diet and perform physical activity through a questionnaire, which may not be an accurate picture of the participant’s diet and physical activity self-management. Although HAPA may be part of the solution to the behavior intention gap, the absence of social factors limits it. Since our study population was low average age, half of them had a university degree, and most of them were from medium to high socio-economic status, the results should be generated precautious.

## Conclusion

The present study showed that the score of healthy diet and physical activity self-management was low in patients who underwent bariatric surgery. Psychological variables associated with HAPA could adequately explain healthy diet behavior and physical activity among the patients. In addition, task and coping self-efficacy, risk perception, and outcome expectancies significantly affected diet and physical activity intention. Using HAPA with special attention to the contribution of the constructs is suggested. Moreover, it is suggested to design interventions with the lens of HAPA constructs to improve healthy diet and physical activity self-management in patients after bariatric surgery.

## Supporting information

S1 Data (XLSX)
